# Molecular barriers to direct cardiac reprogramming

**DOI:** 10.1007/s13238-017-0402-x

**Published:** 2017-04-07

**Authors:** Haley Vaseghi, Jiandong Liu, Li Qian

**Affiliations:** Department of Pathology and Laboratory Medicine, McAllister Heart Institute, University of North Carolina, Chapel Hill, NC 27599 USA

**Keywords:** cardiac reprogramming, myocardial infarction, epigenetics, heart regeneration

## Abstract

Myocardial infarction afflicts close to three quarters of a million Americans annually, resulting in reduced heart function, arrhythmia, and frequently death. Cardiomyocyte death reduces the heart’s pump capacity while the deposition of a non-conductive scar incurs the risk of arrhythmia. Direct cardiac reprogramming emerged as a novel technology to simultaneously reduce scar tissue and generate new cardiomyocytes to restore cardiac function. This technology converts endogenous cardiac fibroblasts directly into induced cardiomyocyte-like cells using a variety of cocktails including transcription factors, microRNAs, and small molecules. Although promising, direct cardiac reprogramming is still in its fledging phase, and numerous barriers have to be overcome prior to its clinical application. This review discusses current findings to optimize reprogramming efficiency, including reprogramming factor cocktails and stoichiometry, epigenetic barriers to cell fate reprogramming, incomplete conversion and residual fibroblast identity, requisite growth factors, and environmental cues. Finally, we address the current challenges and future directions for the field.

## INTRODUCTION

As the leading cause of death in the United States, heart disease accounts for one out of every four mortalities (Heart Disease Fact Sheet CDC, 2016). Contributing to this, every year 735,000 Americans experience a myocardial infarction (Heart Attack Facts & Statistics CDC, 2016) which reduces the heart’s pump capacity due to cardiomyocyte death and increases the risk of arrhythmia from the deposition of non-conductive scar tissue. There are currently three primary approaches to cardiac regeneration as a therapy for myocardial infarction: 1) progenitor cell transplantation, 2) induced proliferation of resident cardiomyocytes, and 3) non-myocyte cell fate reprogramming. The first, progenitor cell therapy, is limited by the low viability and integration of transplanted cells. Multiple clinical trials have demonstrated that the engraftment rate of transplanted cells and the number of cardiomyocytes derived from transplanted progenitors are insufficient to produce a therapeutic effect (Lin and Pu, 2014). However, some clinical benefit is observed due to paracrine signaling from the transplanted progenitors (Lin and Pu, 2014). Identification of the contributing factors will produce the same effect while bypassing transplantation altogether (Lin and Pu, 2014). The second approach, inducing myocyte cell cycle re-entry, is accompanied by both efficacy and safety concerns. Studies have yet to demonstrate that the proposed methods for stimulating cardiomyocyte proliferation can generate a sufficient quantity of new cardiomyocytes to produce a clinically relevant effect (Lin and Pu, 2014). Furthermore, induced proliferation approaches must demonstrate cardiomyocyte-selective stimulation to preclude oncogenesis (Lin and Pu, 2014). The third approach, cell fate reprogramming of endogenous non-myocytes, is addressed in this review.

**Figure 1:**
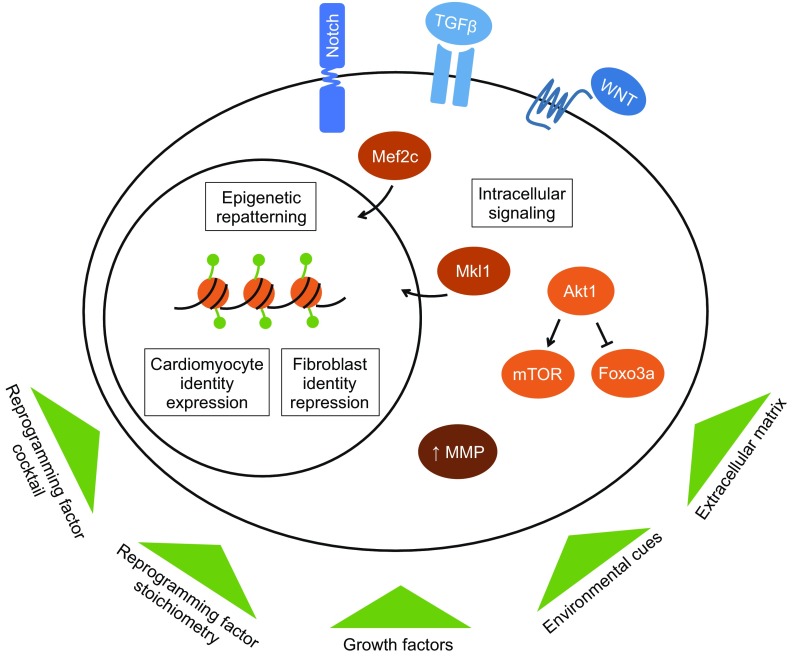
Exogenous factors including reprogramming factor cocktails and stoichiometry and environmental stimuli alter intracellular signaling pathways and epigenetic repatterning to suppress fibroblast gene expression and promote cardiomyocyte gene expression to enhance direct cardiac reprogramming

Cell fate reprogramming confers a dual advantage of reducing scar tissue while simultaneously generating new cardiomyocytes. Following coronary artery ligation, direct cardiac reprogramming reduces scar size in murine hearts by over two-fold (Qian et al., 2012; Song et al., 2012). New fibroblast-derived reprogrammed cardiomyocytes comprise 35% of cardiomyocytes in the infarct and border zones (Qian et al., 2012). These newly reprogrammed cardiomyocytes exhibit integration into the working myocardium, with electrical connectivity to endogenous cardiomyocytes and coordinated contraction (Qian et al., 2012; Song et al., 2012). Concomitant with measurably reducing scar size and generating new cardiomyocytes, direct cardiac reprogramming demonstrates substantial therapeutic benefit. Reprogramming therapy improves ejection fraction, stroke volume, and cardiac output in murine hearts following coronary artery ligation (Qian et al., 2012; Song et al., 2012) and sustains improvement up to 12 weeks after myocardial infarction (Song et al.,^2012^), demonstrating its potential as a therapeutic approach. With the *in situ* regeneration of mature, functional cardiomyocytes and simultaneous reduction in scar tissue, direct cardiac reprogramming has strong potential as a clinical therapy to restore cardiac function following myocardial infarction. Although significant advances have been made, studies have uncovered numerous molecular barriers to the reprogramming process (Fig. 1). This review explores the molecular barriers to cell fate conversion in order to facilitate effective and complete direct cardiac reprogramming.

## REPROGRAMMING FACTOR COCKTAILS

Observation that only a fraction of the starting cell population fully reprograms into functional cardiomyocytes has prompted numerous initiatives to screen candidate reprogramming factors in order to identify cocktails for optimal reprogramming efficiency. Studies have screened cocktails of transcription factors (Song et al., 2012; Ieda et al., 2010; Protze et al., 2012; Christoforou et al., 2013; Addis et al., 2013), microRNAs (Jayawardena et al., 2012 2015), small molecules (Fu et al., 2015; Mohamed et al., 2016), and combinations of the three (Ifkovits et al., 2014; Wang et al., 2014; Muraoka et al., 2014) (Table 1).

**Table 1:** Cocktails for direct cardiac reprogramming of mouse fibroblasts *in vitro* and *in vivo* (*)

*Transcription Factor Cocktails*	
Gata4, Mef2c, Tbx5 (GMT)*	Ieda et al. (2010)
Gata4, Hand 2, Mef2c, Tbx5 (GHMT)*	Song et al. (2012)
Mef2c, Tbx5, Myocd	Protze et al. (2012)
Gata4, Mef2c, Tbx5, Myocd, SRF, Mesp1, SMARCD3	Christoforou et al. (2013)
Hand2, Nxk2.5, Gata4, Mef2c, Tbx5 (HNGMT)	Addis et al. (2013)
*MicroRNA Cocktail*	
miR-1, miR-133, miR-208, miR-499*	Jayawardena et al. (2012)
*Chemical Cocktail*	
CHIR99021, RepSox, Forskolin, VPA, Parnate, TTNPB	Fu et al. (2015)
*Combined Cocktails*	
MicroRNA cocktail + JAK inhibitor I	Jayawardena et al. (2012)
Chemical cocktail + Oct4	Wang et al. (2014)
HNGMT + TGFβ inhibitor	Ifkovits et al. (2014)
GMT + miR-1/miR-133	Muraoka et al. (2014)
GMT + WNT/TGFβ inhibitor	Mohamed et al. (2016)

Direct cardiac reprogramming was first described using a transcription factor cocktail. Ieda et al. screened a pool of fourteen cardiac lineage transcription factors to define a cocktail of three reprogramming factors—Gata4, Mef2c, and Tbx5 (GMT)—that are necessary and sufficient to convert fibroblasts to induced cardiomyocyte-like cells (iCMs) both *in vitro* (Ieda et al., 2010; Wang et al., 2015a) and *in vivo* (Qian et al., 2012; Inagawa et al., 2012; Ma et al., 2015). Subsequently, Song et al. screened six conserved cardiac lineage transcription factors to identify a cocktail of Gata4, Hand2, Mef2c, and Tbx5 (GHMT) that generates almost five-fold more cTnT/αMHC-GFP double positive iCMs than GMT *in vitro* (Song et al., 2012). The GHMT cocktail also generates iCMs in murine hearts *in vivo*, reducing scar size and improving cardiac function following myocardial infarction (Song et al., 2012). While the above mentioned studies employed sequential subtraction of candidate factors and αMHC-GFP reporter expression to screen cocktails, Protze et al. tested all possible triplet combinations of ten reprogramming factors and used quantitate PCR for multiple cardiac markers to identify a three factor reprogramming cocktail of Mef2c, Tbx5, and Myocd that induces expression of a broad range of cardiac genes (Protze et al., 2012). Christoforou et al. furthermore demonstrated that a five or a seven factor cocktail adding Myocd, SRF, Mesp1, and SMARCD3 to the three factor GMT cocktail enhances cTnT mRNA expression by almost five-fold (Christoforou et al., 2013). Finally, Addis et al. employed a calcium reporter to functionally screen candidate factors and define a five factor cocktail of Hand2, Nkx2.5, Gata4, Mef2c, and Tbx5 (HNGMT) for maximally efficient reprogramming of functional iCMs (Addis et al., 2013).

In addition to transcription factor cocktails, microRNA combinations successfully generate reprogrammed iCMs. Jayawardena et al. screened six candidate microRNAs selected for their regulatory role in cardiomyocyte differentiation and development, transfecting cardiac fibroblasts with all 41 permutations of singlet, doublet, and triplet microRNA combinations and screening using quantitative PCR for cardiac markers (Jayawardena et al., 2012). A cocktail of miR-1, miR-133, miR-208, and miR-499 is sufficient to reprogram cardiac fibroblasts both *in vitro* (Jayawardena et al., 2012) and *in vivo* (Jayawardena et al., 2015). The microRNA cocktail generates functional, mature iCMs *in vivo* and reduces scar size and improves cardiac function in murine hearts following myocardial infarction (Jayawardena et al., 2015). The mechanism of miR-1 and miR-133 enhancement of reprogramming is discussed below in the “REPRESSION OF FIBROBLAST IDENTITY” section.

Chemical cocktails for direct cardiac reprogramming have also been developed, circumventing the genetic manipulation associated risks involved in transcription factor and microRNA cocktails. Fu et al. noted the emergence of spontaneously beating cells during iPSC reprogramming using a cocktail of small molecule compounds and developed a two-step reprogramming process to induce and stabilize iCM reprogramming (Fu et al., 2015). This two-step chemical cocktail reprogramming generates beating clusters of iCMs that express cardiac markers, assemble contractile sarcomeres, and display cardiomyocyte-like electrophysical properties without going through a pluripotent stage (Fu et al., 2015).

In addition to pure cocktails of transcription factors, microRNA, or small molecules, combinations of factors produce a synergistic effect for maximal reprogramming efficiency. Wang et al. directly reprogrammed fibroblasts into iCMs without going through a pluripotent intermediate state using a chemical cocktail plus a single transcription factor Oct4 (Wang et al., 2014). Conversely, Ifkovits et al. used a single small molecule TGFβ inhibitor to enhance reprogramming efficiency of the transcription factor cocktail HNGMT by five-fold (Ifkovits et al., 2014). Mohamed et al. found that WNT and TGFβ inhibitors enhance GMT transcription factor cocktail reprogramming by eight-fold (Mohamed et al., 2016). The effects of TGFβ and WNT signaling in reprogramming are discussed below in the “REPRESSION OF FIBROBLAST IDENTITY” and “INTRACELLULAR SIGNALING PATHWAYS” sections. Muraoka et al. found that miR-1 or miR-133 together with the transcription factor cocktail GMT produced a six-fold increase over GMT alone (Muraoka et al., 2014). The mechanism of miR-1 and miR-133 enhancement of reprogramming is also discussed below in the “REPRESSION OF FIBROBLAST IDENTITY” section. Jayawardena et al. used a small molecule compound, JAK inhibitor I, in combination with the microRNA cocktail miR-1, miR-133, miR-208, and miR-499 to increase reprogramming efficiency by ten-fold (Jayawardena et al., 2012).

## REPROGRAMMING FACTOR STOICHIOMETRY

Decades of research in developmental biology have revealed the fine balance of transcription factor expression that is required to initiate and maintain cardiac lineage commitment. However, in cell fate reprogramming, the forced overexpression of reprogramming factors results in crude, artificial transcription factor dosage. Most studies using the standard Gata4, Mef2c, Tbx5 cocktail utilize retroviral delivery of the three factors packaged as separate viruses. Starting cells must take up each of the three individual viruses in order to be reprogrammed, leading to low cell fate conversion rates since only a subset of cells receive all three factors. Individual cells also receive different ratios of the three factors. Stochastically, only a small fraction of cells receive the optimal reprogramming factor ratio and dose for cell fate reprogramming.

To address reprogramming factor stoichiometry, our lab determined the optimal ratio of the three transcription factors for efficient reprogramming by creating six polycistronic constructs with the three factors in every possible splice order separated by 2A peptide cleavage sites (Wang et al., 2015a). Splice order dictates protein expression level, with highest protein expression for the factor in the first position and lower protein expression for the factors in the second and third positions (Wang et al., 2015a). Only the Mef2c-Gata4-Tbx5 (MGT) and Mef2c-Tbx5-Gata4 (MTG) constructs, which have Mef2c in the first position, increase reprogramming efficiency compared to infection with three separate Gata4, Mef2c, and Tbx5 viruses (Wang et al., 2015a). The other four constructs decreased αMHC-GFP iCM reporter expression compared to reprogramming with separate viruses (Wang et al., 2015a). The highest reporter expression was achieved using the MGT splice order with concomitantly higher levels of Mef2c and lower levels of Gata4 and Tbx5 (Wang et al., 2015a). The optimal polycistronic MGT vector improved both αMHC-GFP reporter expression and cTnT cardiac marker expression compared to separate Gata4, Mef2c, Tbx5 virus reprogramming (Wang et al., 2015a). The polycistronic MGT vector generated reprogrammed cells that formed iCM clusters, assembled cTnT and α-Actinin positive sarcomeres, expressed the gap junction protein Connexin 43, exhibited calcium flux, and contracted spontaneously (Wang et al., 2015a). Molecular characterization also shows that the MGT and MTG polycistronic vectors induce higher expression of cardiomyocyte genes and lower expression of cardiac stress genes *Nppa* and *Nppb* than reprogramming with separate viruses (Wang et al., 2015a). An optimized protocol for reprogramming using the polycistronic MGT system was described as an additional resource (Wang et al., 2015b).

We also demonstrated that optimal reprogramming factor stoichiometry using the polycistronic MGT vector improves reprogramming efficiency *in vivo* in a mouse model of myocardial infarction (Ma et al., 2015). Using Periostin-Cre; R26R-lacZ genetic lineage tracing, cells of fibroblast origin were irreversibly marked with β-galactosidase to identify iCMs generated from fibroblasts (Ma et al., 2015). *In vivo* reprogramming with polycistronic MGT following coronary artery ligation increases the number of α-Actinin/β-galactosidase double positive iCMs compared to reprogramming with separate Gata4, Mef2c, Tbx5 viruses (Ma et al., 2015). Additionally, *in vivo* reprogramming with the polycistronic MGT vector produces greater functional improvement in fractional shortening and ejection fraction and reduces scar size following myocardial infarction than reprogramming with separate viruses (Ma et al., 2015). Optimal stoichiometry of the three transcription factors using a polycistronic vector increases the efficiency of *in vivo* reprogramming and further improves cardiac function following myocardial infarction (Ma et al., 2015).

In addition to defining the optimal reprogramming factor stoichiometry, we created an inducible system where polycistronic MGT expression is both temporally and quantitatively regulated through the administration of doxycycline (Vaseghi et al., 2016). Using the TetOn inducible gene expression system, reprogramming factor expression is tightly regulated by the presence of doxycycline in the cell culture media (Vaseghi et al., 2016). Additionally, by titrating the doxycycline concentration, reprogramming factor expression level and dosage can be controlled (Vaseghi et al., 2016).

Two other *in vivo* studies also created polycistronic reprogramming vectors. Inagawa et al. placed the reprogramming factors in the order Tbx5-Mef2c-Gata4 (Inagawa et al., 2012), while Mathison et al. used the order Gata4-Mef2c-Tbx5 (Mathison et al., 2014). Both studies demonstrate a modest increase in reprogramming efficiency using their single polycistronic vector compared to three separate viruses, but the improvement is only incremental due to the non-optimal dosage of the reprogramming factors.

Inagawa et al. found no difference in the proportion of α-Actinin positive reprogrammed cells to infected cells between their polycistronic TMG vector and separate viruses but did see the proportion of α-Actinin positive cells that had assembled sarcomeres double from 15% to 30% of infected cells (Inagawa et al., 2012). Mathison et al. demonstrated increased numbers of reprogrammed cells *in vitro* and improved cardiac function *in vivo* using their GMT polycistronic vector (Mathison et al., 2014). The incremental improvement demonstrated by these polycistronic systems is possibly due to the increased delivery of all three reprogramming factors to the starting cell population, although the optimal dosage of the reprogramming factors was not considered.

## EPIGENETIC BARRIERS TO DIRECT CARDIAC REPROGRAMMING

In addition to reprogramming factor dosage and timing, the epigenetic regulation of gene expression programs is critical to cell lineage commitment. Highlighting the role of epigenetic modulation in cell fate determination and conversion, Takeuchi and Bruneau directed ectopic differentiation of embryonic mouse mesoderm into beating cardiomyocytes using transient transfection of the cardiac transcription factor Gata4 and the cardiac specific chromatin remodeling complex subunit, Baf60c (Takeuchi and Bruneau, 2009). Baf60c potentiates the binding of Gata4 to DNA at cardiac loci to turn on ectopic cardiac gene expression (Takeuchi and Bruneau, 2009). Transfection of the transcription factor Tbx5 in addition to Gata4 and Baf60c promotes the complete differentiation of transfected cells into beating cardiomyocytes (Takeuchi and Bruneau, 2009). The transfected cells begin beating even before the endogenous heart field starts to form (Takeuchi and Bruneau, 2009). Baf60c is one of the minimal requirements for this ectopic cardiac differentiation of the embryonic mesoderm, demonstrating that chromatin remodeling plays a crucial role in transdifferentiation and cardiac fate acquisition. Cellular reprogramming necessarily involves changes to the epigenetic landscape. Epigenetic marks must be erased and re-written to alter chromatin structure and gene expression patterns during reprogramming as the fibroblast signature is repressed and a cardiomyocyte gene expression program is activated. Recent studies have demonstrated that epigenetic manipulation can potentiate the reprogramming process.

Epigenetic regulation of gene transcription is mediated through histone post-translational modification, including histone acetylation. Studies in cardiac development suggest that histone deacetylase (HDAC) inhibition directs cells toward a cardiac lineage fate (Karamboulas et al., 2006; Chen et al., 2011). Overexpression of HDAC4, a class IIa HDAC, inhibits cardiac muscle development, while class IIa HDAC inhibition restores cardiomyogenesis (Karamboulas et al., 2006). Commitment to the cardiac lineage is accompanied by the upregulation of Gata4, Mef2c, and other key cardiac transcription factors (Karamboulas et al., 2006); however, class IIa HDACs repress cardiac transcription factors, including Gata4 and Mef2 family transcription factors (McKinsey and Olson 2004; Miska et al., 1999). Consequently, HDAC inhibition promotes cardiomyogenesis and upregulates the expression of cardiac transcription factors Gata4, Mef2c, and Nkx2.5, among others (Chen et al., 2011). Simultaneous induction of reprogramming factor expression and HDAC inhibition using valproic acid, a non-specific HDAC inhibitor, enhances direct cardiac reprogramming, increasing the proportion of cTnT or α-Actinin positive reprogrammed cells by two-fold (Christoforou et al., 2013). These findings suggest that HDAC inhibition may enhance direct cardiac reprogramming by epigenetically priming cells for cardiac fate acquisition.

Widespread epigenetic repatterning occurs during direct cardiac reprogramming. Zhao et al. performed chromatin immunoprecipitation sequencing of H3K4me2, which marks the promoters and enhancers of transcriptionally active genes, and demonstrated that one week after GHMT cocktail reprogramming 47% of the H3K4me2 peaks had shifted to align with those of primary cardiomyocytes (Zhao et al., 2015). Our lab demonstrated that trimethylation of lysine 27 on histone 3 (H3K27me3), a repressive epigenetic mark, is depleted and trimethylation of lysine 4 on histone 3 (H3K4me3), an activating histone modification, is enriched at cardiac promoters early in GMT cocktail reprogramming and is accompanied by a rapid increase in cardiac gene mRNA expression (Liu et al., 2016a). This early activation of the cardiomyocyte gene expression program is later followed by the increase of H3K27me3 and decrease of H3K4me3 at fibroblast loci and a concomitant decrease in fibroblast gene mRNA expression (Liu et al. 2016a). These findings suggest that reprogramming activates cardiac gene expression first, followed by later repression of fibroblast gene expression. Similarly, Dal-Pra et al. demonstrated that H3K27 demethylation is required for the induction of cardiac gene expression during microRNA cocktail reprogramming (Dal-Pra et al., 2017). Reprogramming with a miR-1, miR-133, miR-208, miR-499 microRNA cocktail alters H3K27 methyltransferase and demethylase expression, inducing a 40% and 50% increase in expression of the two H3K27 demethylases Kdm6B and Kdm6A respectively and a 50% decrease in expression of the H3K27 methyltransferase Ezh2 at both the mRNA and protein level (Dal-Pra et al., 2017). Consequently, reprogramming reduces global H3K27me3 by 40% (Dal-Pra et al., 2017). Specifically, promoters of cardiac transcription factors Tbx5, Mef2c, and Gata4 exhibit a 50% reduction in H3K27me3 (Dal-Pra et al., 2017). Pharmacologic inhibition of H3K27 methyltransferase activity reduces H3K27me3 by 30% and increases gene and protein expression of cardiac markers between two- to eight-fold irrespective of microRNA cocktail reprogramming (Dal-Pra et al., 2017). Conversely, knockdown of H3K27 demethylase activity inhibits the induction of cardiac marker expression during microRNA cocktail reprogramming (Dal-Pra et al., 2017). These findings demonstrate that H3K27 demethylation and de-repression of cardiac loci is essential for direct cardiac reprogramming.

Given that extensive repatterning of histone modifications occurs during the reprogramming process, modulation of the deposition and removal of histone modifications may promote greater reprogramming efficiency. Hirai et al. used a small molecule inhibitor of Ezh2, the catalytic component of the PRC2 complex catalyzing H3K27me2/3, to demonstrate that Ezh2 inhibition early in reprogramming increases reprogramming efficiency (Hirai and Kikyo, 2014). The authors also report that late inhibition of the methyltransferase G9a, which catalyzes H3K9me1/2, increases reprogramming efficiency (Hirai and Kikyo, 2014). Conversely, Ifkovits et al. found that pre-treatment of fibroblasts with a G9a histone methyltransferase inhibitor reduced reprogramming efficiency (Ifkovits et al., 2014), demonstrating that the timing of histone methyltransferase inhibition is crucial for its effect on reprogramming. Hirai et al. observed that only specific time windows of drug administration were sufficient to promote an increase in reprogrammed cells and that inhibition at other times resulted in no effect or a decrease in reprogramming efficiency (Hirai and Kikyo, 2014). The two histone methyltransferases inhibited by Hirai et al., Ezh2 and G9a, require inhibition at different times to promote increased reprogramming (Hirai and Kikyo, 2014). While early inhibition of Ezh2 resulted in maximal generation of iCM clusters, late inhibition of G9a promoted iCM generation (Hirai and Kikyo, 2014), indicating that the timing of histone methyltransferase inhibition is critical to enhancing reprogramming.

To identify key epigenetic barriers to the reprogramming process, we conducted a comprehensive loss-of-function screen of epigenetic modifying factors and identified Bmi1 as a critical epigenetic inhibitor of reprogramming (Zhou et al., 2016). Bmi1 is a polycomb group protein that directly binds to cardiac loci and suppresses expression of cardiac genes (Zhou et al., 2016). Depleting Bmi1 increases H3K4me3 and reduces H2AK119ub at cardiac loci and subsequently de-represses cardiac gene expression, priming fibroblasts for reprogramming (Zhou et al., 2016). Bmi1 knockdown plays a role to enhance cardiac fate acquisition early in the reprogramming process, as late Bmi1 depletion does not affect reprogramming (Zhou et al., 2016). Bmi1 also directly binds to the regulatory region and modulates the expression of Gata4 (Zhou et al., 2016). The inhibition of Bmi1 de-represses endogenous Gata4 expression and can therefore replace exogenous Gata4 in the Gata4, Mef2c, Tbx5 reprogramming cocktail (Zhou et al., 2016), permitting two factor-mediated iCM reprogramming to be possible.

In a similar approach, Liu et al. conducted a gain-of-function screen of cardiac development epigenetic modifiers and transcription factors and identified the H3K4 methyltransferase Mll1 as a barrier to direct cardiac reprogramming (Liu et al., 2016b). Pharmacologic inhibition of Mll1 improves both iCM generation with a 1.5-fold increase in αMHC-GFP reporter expression and iCM maturation with increased sarcomere assembly and spontaneous beating (Liu et al., 2016b). Inhibition of Mll1 directs cardiomyocyte cell fate specification by suppressing adipocyte lineage transdifferentiation (Liu et al., 2016b). Mll1 inhibition prevents MGT-mediated upregulation of adipocyte genes and adipocyte formation as indicated by Oil Red O staining (Liu et al., 2016b). The adipocyte associated gene *Ebf1* is a key target of Mll1, and the reduction in *Ebf1* expression caused by Mll1 inhibition mediates the observed increase in reprogramming efficiency (Liu et al., 2016b).

## REPRESSION OF FIBROBLAST IDENTITY

Direct cardiac reprogramming is accomplished through the activation of cardiac gene expression but must also be accompanied by complete silencing of the original fibroblast signature. Characterization of epigenetic repatterning during reprogramming by Liu et al. demonstrates a gradual repression of fibroblast loci (Liu et al., 2016a). Chromatin immunoprecipitation followed by real time PCR reveals late deposition of the repressive H3K27me3 histone modification at fibroblast marker gene promoters and fibroblast-enriched transcription factor promoters with a concomitant decrease in mRNA expression (Liu et al., 2016a).

Maintenance of residual fibroblast gene expression presents a roadblock to the successful reprogramming of functionally mature cardiomyocytes. MicroRNAs miR-1 and miR-133 enhance direct cardiac reprogramming by suppressing fibrotic gene expression (Muraoka et al., 2014; Zhao et al., 2015). Overexpression of miR-1 and miR-133 together with reprogramming cocktails generates more spontaneously beating iCMs faster than reprogramming cocktails alone (Muraoka et al., 2014; Zhao et al., 2015). Reprogramming with miR-133 also generates more iCMs exhibiting spontaneous calcium oscillations than transcription factor cocktails alone (Muraoka et al., 2014). A combination of both miR-1 and miR-133 with transcription factor reprogramming cocktails was the most efficient treatment for generating spontaneously beating iCMs (Zhao et al., 2015). MiR-133 overexpression represses fibroblast gene expression through the suppression of Snai1 (Muraoka et al., 2014). Snai1 knockdown suppresses fibroblast gene expression and promotes cardiac gene expression in reprogramming, while Snai1 overexpression maintains fibroblast gene expression and inhibits the development of spontaneous beating in iCMs (Muraoka et al., 2014). These studies indicate that microRNA repression of fibroblast gene expression improves reprogramming speed and iCM functional maturity.

In another approach to erasing fibroblast identity, studies have used inhibition of pro-fibrotic signaling pathways to enhance reprogramming efficiency (Ifkovits et al., 2014; Zhao et al., 2015). Silencing pro-fibrotic transforming growth factor beta (TGFβ) signaling and Rho-associated kinase (ROCK) signaling increases iCM conversion numbers and speed (Ifkovits et al., 2014; Zhao et al., 2015). Conversely, overexpression of these pathways inhibits the reprogramming process (Ifkovits et al., 2014; Zhao et al., 2015). Maintenance of pro-fibrotic signaling leads to incompletely converted cells; however, reprogramming factor expression is sufficient to activate pro-fibrotic signaling, which must be subsequently suppressed for successful conversion (Zhao et al., 2015). Early administration or pre-treatment with a TGFβ silences pro-fibrotic signaling to enhance reprogramming efficiency (Mohamed et al. 2016; Ifkovits et al., 2014). In conjunction with miR-1 and miR-133 overexpression, ROCK or TGFβ further represses fibroblast gene expression and increases reprogramming efficiency and speed, suggesting a synergistic barrier to reprogramming between pro-fibrotic signaling and microRNA fibroblast gene regulation (Zhao et al., 2015). Similarly, dual inhibition of TGFβ Wnt signaling improves the quantity, maturation, and speed of reprogramming (Mohamed et al., 2016). Early TGFβ inhibition followed shortly by Wnt inhibition generates an eight-fold increase in iCMs (Mohamed et al., 2016). These iCMs exhibit accelerated reprogramming with beating cells observed as early as one week after reprogramming (Mohamed et al., 2016). TGFβ and Wnt inhibition during reprogramming produces iCMs that are transcriptionally more similar to adult cardiomyocytes than iCMs generated in the absence of inhibitors (Mohamed et al., 2016). Inhibition of TGFβ and Wnt signaling affects chromatin accessibility (Mohamed et al., 2016). Together, the dual inhibition of TGFβ and Wnt signaling increases expression of mature cardiomyocyte markers including ion channels, calcium handling genes, and components of fatty acid metabolism (Mohamed et al., 2016). These studies demonstrate that residual fibroblast signature is a barrier to complete cell fate conversion and that repressing fibroblast gene expression enhances reprogramming *in vitro*.

Mohamed et al. demonstrate that repression of residual fibroblast signature using TGFβ and Wnt inhibition also enhances reprogramming and cardiac function *in vivo* (Mohamed et al. 2016). In conjunction with TGFβ and Wnt inhibition, GMT transcription factor cocktail reprogramming increases ejection fraction, stroke volume, and cardiac output as early as one week post coronary artery ligation (Mohamed et al., 2016). Histologic sections reveal that TGFβ and Wnt inhibition reduces scar size and produces thicker bands of fibroblast-derived reprogrammed iCMs re-muscularizing the infarct region (Mohamed et al., 2016). Lineage tracing indicates that TGFβ and Wnt inhibition during reprogramming produces a five-fold increase in iCM generation compared to reprogramming without inhibitors (Mohamed et al., 2016). TGFβ and Wnt inhibition also generates iCMs that are functionally more mature, with calcium and contraction kinetics more similar to adult cardiomyocytes (Mohamed et al., 2016).

## INTRACELLULAR SIGNALING PATHWAYS

Recent studies have examined the effect of intracellular signaling pathways on direct cardiac reprogramming. Zhou et al. modulated intracellular signaling pathways by screening a library of 192 protein kinases to assess the effect on GHMT transcription factor reprogramming (Zhou et al., 2015). Akt1 activation increases reprogramming efficiency and produces iCMs with a more mature cardiomyocyte phenotype, exhibiting an increase in calcium flux, spontaneous beating, polynucleation, cellular hypertrophy, mitochondrial function, cardiac marker expression, and sarcomere assembly (Zhou et al., 2015). Akt1 does not enhance the expression of the GHMT reprogramming factors (Zhou et al., 2015). Rather, Akt1 functions through its downstream targets, activating mTOR and inhibiting Foxo3a which have roles in the regulation of mitochondrial metabolism, myocyte development, and gene expression (Zhou et al., 2015).

Abad et al. screened seven small molecule compounds with a demonstrated role in iPSC reprogramming and found that the Notch inhibitor DAPT enhances GHMT transcription factor cocktail reprogramming (Abad et al., 2017). Notch pathway signaling plays an important role in cardiac development by regulating cardiomyocyte differentiation and proliferation (Abad et al.,^2017^). Non-canonical Notch signaling blocks the binding of transcription factor Mef2c to promoter regions (Abad et al., 2017). In the context of iCM reprogramming, Notch inhibition acts in coordination with Akt1 to increase the acquisition of a mature cardiomyocyte phenotype, demonstrated by increased calcium flux, sarcomere assembly, and spontaneous beating (Abad et al., 2017). GHMT reprogramming with Akt1 activation and Notch inhibition generated up to 70% conversion efficiency with 45% of the reprogrammed iCMs exhibiting spontaneous beating (Abad et al., 2017). Notch inhibition modulates transcriptional programs involved in cardiomyocyte differentiation and development by increasing the binding of transcription factor Mef2c to cardiac loci promoters (Abad et al., 2017).

Mohamed et al. screened a library of 5,500 small molecule compounds in an unbiased, high throughput approach to determine cell signaling pathways that modulate reprogramming and identified the WNT and TGFβ signaling pathways as barriers to reprogramming (Mohamed et al., 2016). Inhibiting both pathways improves GMT transcription factor cocktail reprogramming *in vitro* and *in vivo* (Mohamed et al., 2016). TGFβ/Wnt inhibition enhances reprogramming efficiency and speed *in vitro* (Mohamed et al., 2016). GMT reprogramming with both inhibitors produces 30% αMHC-GFP reporter expression, while only one inhibitor produces 15% and no inhibitors produces only 4% (Mohamed et al., 2016). GMT reprogramming with both inhibitors generates beating iCMs within one week, while only one inhibitor requires three weeks and no inhibitors requires six to eight weeks (Mohamed et al., 2016). TGFβ/Wnt inhibition also enhances reprogramming and cardiac function *in vivo* (Mohamed et al., 2016) (See “REPRESSION OF FIBROBLAST IDENTITY”). RNA sequencing reveals that iCMs reprogrammed in the presence of the TGFβ inhibitor downregulate fibrotic and extracellular matrix associated genes, while iCMs reprogrammed in the presence of the WNT inhibitor downregulate genes affecting chromatin modulation, nucleosome organization, and DNA packaging.

## GROWTH FACTORS

Multiple studies have noted greater conversion efficiency or more complete functional maturation of iCMs reprogrammed *in vivo* over *in vitro* (Qian et al., 2012; Song et al., 2012; Inagawa et al., 2012; Ma et al., 2015). *In vivo* reprogrammed iCMs are more similar to endogenous cardiomyocytes than *in vitro* reprogrammed iCMs. This suggests that unidentified extrinsic factors in the *in vivo* microenvironment such as topographic cues, mechanical forces, growth factors, cytokines, or paracrine signaling play an important role in promoting iCM maturation.

Lack of requisite growth factors constitutes a barrier to reprogramming. Yamakawa et al. noted that under serum-based culture conditions, *in vitro* reprogramming generated incompletely converted, immature iCMs (Yamakawa et al., 2015). Although many reporter positive cells were observed at early time points, few remained marker positive after four weeks of culture (Yamakawa et al., 2015). The authors screened eight cardiogenic compounds to create a serum-free cell culture media containing fibroblast growth factor (FGF) 2, FGF10, and vascular endothelial growth factor (VEGF) that greatly enhances the *in vitro* generation of functionally mature iCMs that contract spontaneously and exhibit calcium oscillations (Yamakawa et al., 2015). The optimized media (FFV) increases the maturity of reprogrammed cells by activating cardiac transcriptional regulators, the p38 MAPK pathway, and the PI3K/AKT pathway (Yamakawa et al., 2015). Additionally, reprogramming with Mef2c and Tbx5 in FFV media upregulates endogenous Gata4 expression and removes the requirement for exogenous Gata4 expression as a reprogramming factor (Yamakawa et al., 2015), similarly to our findings (Zhou et al., 2016). The growth factors in FFV media are critical for late stage maturation but do not affect early reprogramming events (Yamakawa et al., 2015). Christoforou et al. also observed that iCMs cultured in high serum media fail to assemble α-Actinin or cTnT positive sarcomeres (Christoforou et al., 2013). Use of low serum growth media increases the assembly of α-Actinin/cTnT double positive sarcomeres (Christoforou et al., 2013). However, reprogrammed cells lose striated sarcomere staining over time. By day 30 most double positive cells do not exhibit organized sarcomeres (Christoforou et al., 2013). These studies indicate that the growth factors in serum-free media help promote iCM maturation and sarcomere assembly.

## ENVIRONMENTAL CUES

In addition to growth factors, environmental cues are important in developing the functional maturity of iCMs. Cultured cardiomyocytes respond differently to the stiffness of the *in vitro* substrate (Chopra et al., 2012). Polyacrylamide gels with a stiffness between 10–30 kPa favor cardiomyocytes with a spread and elongated morphology that form well organized, polarized sarcomeres. However, stiff substrates produce cells with F-actin stress fibers that lack organized sarcomeres, while soft substrates produce cells with rounded morphology and disorganized sarcomeres. These findings suggest that the cardiomyocyte cytoskeleton remodels based on substrate stiffness (Chopra et al., 2012). However, GMT transcription factor cocktail reprogramming of adult tail tip fibroblasts on substrates of 1, 21, and 62 kPa does not have an effect on reprogramming efficiency even though variation in substrate stiffness successfully induces a range of morphologies (Sia et al., 2016). Culturing reprogramming cells under conditions of periodic uniaxial stretch also fails to increase reprogramming efficiency, although cells orient in response (Sia et al., 2016).

While direct cardiac reprogramming is unaffected by *in vitro* substrate stiffness or mechanical stretch, reprogramming does respond to other topographical cues. Morez et al. demonstrated that the forward programming of cardiac progenitor cells using the cardiac lineage transcription factor cocktail Myocardin, Tbx5, and Mef2c is enhanced by topographical cues which modulate histone acetylation (Morez et al., 2015). Sca1^+^ adult progenitor cells were reprogrammed on flat or microgrooved collagen I coated polydimethylsiloxane substrates. Reprogramming efficiency and sarcomere assembly are enhanced on microgrooved substrates compared to flat substrates (Morez et al., 2015). Culture on microgrooved substrates increases histone 3 acetylation in differentiating cells (Morez et al., 2015). Treatment with the histone deacetylase inhibitor VPA produces a similar increase in reprogramming efficiency and increase in histone 3 acetylation in cells reprogrammed on a flat substrate (Morez et al., 2015). VPA treatment does not produce an additive effect on the reprogramming efficiency of cells cultured on microgrooved substrates, indicating that culture on microgrooved substrates increases reprogramming through histone acetylation (Morez et al., 2015). Culturing on microgrooved substrates also significantly enhances iCM sarcomere assembly compared to flat substrates (Morez et al., 2015). Unlike reprogramming efficiency, sarcomere organization is independent of histone 3 acetylation (Morez et al., 2015). These results indicate that topographical cues improve cardiomyocyte reprogramming efficiency and maturation (Morez et al. 2015). Furthermore, Sia et al. demonstrated that GMT reprogrammed adult tail tip fibroblasts cultured on a microgrooved substrate show increased reprogramming and beating through a histone acetylation and transcriptional activation mechanism (Sia et al., 2016). Cells cultured in microgrooves align along the grooves and exhibit an elongated morphology (Sia et al., 2016). Reprogramming in microgrooves generates two-fold more cTnT positive, sarcomere positive, and beating iCMs than reprogramming on flat surfaces (Sia et al., 2016). Microgroove cultured iCMs have 1.5-fold higher nuclear localization of the mechanosensitive transcription factor Mkl1 than iCMs cultured on flat surfaces (Sia et al., 2016). Blebbistatin treatment prevents Mkl1 nuclear localization and reduces the reprogramming yield of iCMs on microgrooved surfaces to that of flat surfaces (Sia et al., 2016). Jasplakinolide and Cytochalasin D promote Mkl1 nuclear localization and increase the yield of iCMs cultured on flat surfaces to that of grooved surfaces (Sia et al., 2016). However, overexpressing Mkl1 during reprogramming on flat surfaces only partially accounts for the increase in reprogramming seen on grooved surfaces (Sia et al., 2016). Consistent with the findings of Morez et al., Sia et al. showed that culturing on microgrooves increases histone 3 acetylation (Sia et al., 2016). Simultaneous VPA HDAC inhibition and Mkl1 overexpression completely account for the increase in reprogramming on grooved surfaces (Sia et al., 2016).

The identity and composition of the extracellular matrix also impacts cardiomyocyte phenotype and reprogramming. Substrate adhesive ligands alter cardiomyocyte sarcomere organization and maturation through integrin signaling. Sarcomeres are well organized and polarized when cardiomyocytes are cultured on fibronectin coated polyacrylamide substrates but not on collagen I coated polyacrylamide (Chopra et al., 2012). Culturing cardiomyocytes on hyaluronic acid instead of polyacrylamide gels partially removes cardiomyocyte dependence on substrate stiffness and allows cells to organize mature sarcomeres on softer substrates (Chopra et al., 2012). These findings suggest that the cardiomyocyte cytoskeleton remodels based on adhesive ligand signaling (Chopra et al., 2012). Consequently, extracellular matrix composition also influences cardiac reprogramming (Kong et al., 2013). Using an indirect reprogramming method that employs de-differentiation followed by directed cardiac differentiation, Kong et al. compared reprograming efficiency on hydrogels incorporating Matrigel, collagen I, or fibrin extracellular matrix proteins (Kong et al., 2013). Reprogramming on fibrin gels yields the greatest number of contractile cardiomyocyte colonies, and supplementation with ascorbic acid, which promotes cellular collagen synthesis, increases contractile colony size (Kong et al., 2013). Contractile colonies stain positive for collagen while non-contractile colonies are negative (Kong et al., 2013). Furthermore, the addition of collagen I to fibrin hydrogels promotes cardiac differentiation and increases the generation of contractile colonies (Kong et al., 2013). These findings demonstrate that the composition of extracellular matrix proteins for *in vitro* cell culture substrates directly alters reprogramming efficiency and maturity.

To more accurately mimic environmental stimuli *in vivo*, Li et al. cultured reprogramming iCMs in a 3D fibrin hydrogel and demonstrated that 3D culture enhances direct cardiac reprogramming (Li et al., 2016). Compared with 2D culture, 3D hydrogel culture increases cardiac gene expression and cTnT and α-Actinin immunostaining in both microRNA reprogramming cocktail and control microRNA conditions (Li et al., 2016). While microRNA cocktail reprogramming on traditional 2D tissue culture plates produces a five-fold increase in αMHC-CFP reporter expression, reprogramming in 3D fibrin hydrogels generates a twenty-fold increase in reporter expression (Li et al., 2016). Fibrin hydrogel 3D culture also increases matrix metalloproteinase (MMP) expression (Li et al., 2016). Broad spectrum pharmacological inhibition of MMP activity abolishes the increase in reprogramming from fibrin hydrogel 3D culture, indicating that 3D culture enhances reprogramming through a MMP-mediated mechanism (Li et al., 2016). The role of MMPs in enhancing direct cardiac reprogramming in 3D culture suggests that the upregulation of MMPs in infarcted hearts could be a contributing factor to the greater reprogramming efficiency *in vivo* compared to *in vitro* (Li et al., 2016).

Manipulation of *in vivo* conditions also has the potential to improve reprogramming. Promoting angiogenesis through preconditioning with VEGF increases *in vivo* reprogramming efficiency and improves therapeutic restoration of cardiac function (Mathison et al., 2012). Pro-angiogenic VEGF treatment increases the vascularization of the infarct zone in rat hearts following myocardial infarction (Mathison et al., 2012). VEGF preconditioning also increases the number of *Myh7* positive cardiomyocytes in the infarct zone of GMT treated hearts and increases ejection fraction by four-fold (Mathison et al., 2012). Promoting fibroblast activation and migration through thymosin b4 treatment also enhances *in vivo* reprogramming efficiency (Qian et al., 2012). Thymosin b4 injection increases fibroblast proliferation in mouse hearts following myocardial infarction (Qian et al., 2012). Thymosin b4 treatment in conjunction with GMT reprogramming increases the generation of iCMs, improves cardiac function, and reduces scar size (Qian et al., 2012).

## FUTURE DIRECTION

Additional research is required to translate direct cardiac reprogramming into a clinical therapy. Necessary steps include continued basic research, research in large animal models, improvement in human reprogramming, and bioengineering of delivery mechanisms.

A better understanding is needed of the mechanism of late stage reprogramming events and iCM maturation. Research in this area is currently hindered by inefficiency in the reprogramming process. Asynchronous, heterogeneous cell populations produce low rates of fully reprogrammed cells making it difficult to acquire sufficient cell numbers to study late stage reprogramming events. Early stage studies are aided by the comparative synchrony of cells early in the reprogramming process that provides a large sample population.

Research in large animal models is also required to move direct cardiac reprogramming toward clinical application. The efficiency of housing, breeding, and handling rodents has made them the most widely used animal models in biomedical research. Additionally, a wealth of tools has been developed specifically for murine research, including imaging techniques, *in vivo* monitoring systems, and genetic manipulation, making the mouse a particularly productive model. However, the mouse exhibits significant cardiovascular differences compared to humans. In addition to obvious differences such as small size and short lifespan, mice differ from humans in a range of anatomical, physiological, energetic, electrophysical, and mechanical properties that include heart rate, coronary artery structure, and contraction/relaxation kinetics. Large animal models such as the dog, sheep, or pig have greater physiologic resemblance to humans with similar body size, heart size, and heart rate. In fact, physiological similarities between pigs and humans are close enough to make the pig an ideal xenotransplant donor. Recent progress in genetic manipulation of pigs will contribute to the use of the pig as a cardiovascular disease model.

Although significant progress has been achieved in uncovering the molecular barriers to direct reprogramming in mice, research in reprogramming human cells lags far behind. Reprogramming human fibroblasts requires the addition of extra factors but yields far lower conversion efficiency. Spontaneously beating cells are rare, indicating that more work is required to translate findings from the mouse to human and uncover undiscovered molecular barriers in human reprogramming.

Finally, a safe and efficient delivery system is required for the translation of direct cardiac reprogramming to clinical use. Current *in vivo* research uses direct injection of retroviral vectors into the infarct zone. While conceivably, direct cardiac injection could be achieved in some myocardial infarct patients during coronary bypass surgery, a non-invasive delivery method is preferable. Additionally, retroviral vectors integrate into the host cell genome, incurring the risk of gene disruption and cellular transformation. The ideal delivery vector would be non-integrating with high transfection efficiency, specificity for the target cell type, and adequate capacity to accommodate multiple reprogramming factors. Adenoviruses are promising viral vectors for the delivery of reprogramming factors. The most commonly employed vector in clinical trials, adenoviruses are non-integrating, with large capacity and high transduction efficiency. Additionally, recent research using small molecules to achieve reprogramming and developments in nanoparticle delivery systems offer potential alternatives to viral vector reprogramming factor delivery.
